# Impact of COVID-19 on Mobile Technology use in adults in the United States

**DOI:** 10.12688/f1000research.136724.1

**Published:** 2023-10-18

**Authors:** Yi Lin, Graham D. Rowles, Arnold J. Stromberg, Rola S. Zeidan, Robert T. Mankowski

**Affiliations:** 1 Physiology and Aging, University of Florida, Gainesville, Florida, 32611, USA; 2 Graduate Center for Gerontology, University of Kentucky, Lexington, Kentucky, 40508, USA; 3 Department of Statistics, University of Kentucky, Lexington, Kentucky, 40508, USA

**Keywords:** behavior, perception, perspective, choice of function, decision to use, user need analysis, technology acceptance model

## Abstract

**Background:** Mobile technology (MT) has become essential in receiving information and services during the COVID-19 pandemic. Imposed quarantines could have led to varying adaptations of MT use. This study explored how COVID-19 impacted behavior, perception, and attitudes toward MT use in the United States.

**Methods:** We distributed a pilot-tested survey online. Participants were MT users ≥ 35 years old. All participants responded based on their recalled experience of using MT before COVID-19 and their recent experience during COVID-19. Data analysis involved descriptive statistics and the Wilcoxon signed-rank test.

**Results:** The average age of the 1212 participants was 56.1±12.2 years (55% female). Daily use frequency (from ≤3 to ≥4 hours/day) and perceived necessity (from some need to a strong need) during COVID-19 significantly increased (p<0.001) compared to before COVID-19. There was a significant increase (p<0.001) in video calls/meetings, online education, grocery/food delivery, and ordering taxi/car during COVID-19 compared to before. Participants increased (p≤0.001) their attention to the physical, social, and emotional benefits of using MT during the pandemic. COVID-19 increased MT use and acceptance in the United States.

**Conclusion:** The knowledge gained from this study will help remove barriers to using and accepting MT and provide directions for MT development in middle-aged and older populations.

## Introduction

COVID-19 was a disease caused by the SARS-CoV-2 virus and has become a worldwide pandemic since 2020. The SARS-CoV-2 virus was easily spread from person to person ( Rothan & Byrareddy, 2020), which led to long-lasting quarantines and the need for spatial distancing. Federal and local governments and public health professionals advised people to stay home, reduce their physical contact with others, and reduce their frequency of going to public places. The associated change in lifestyles directly increased the use of digital technologies such as next-generation telecommunication networks and artificial intelligence ( Ting *et al.*, 2020). Research indicated that using digital technologies could be a solution for pandemic preparedness and response; for instance, healthcare or surveillance technologies could be helpful for pandemic management ( Whitelaw *et al.*, 2020). Researchers suggested investments in mobile applications as valuable tools for developing digital health and enhancing communication ( Torous *et al.*, 2020). At least 114 mobile applications were developed and used during the COVID-19 pandemic ( Collado-Borrell *et al.*, 2020), which reflects the significance of mobile technology (MT) use as a coping strategy.

With its portable features and convenience, MT has become a primary source of information, knowledge, services, and connections ( Bhavya & Sambhav, 2020). MTs (e.g., smartphones, tablets, or smartwatches) differ from traditional ones. Smartphones, for example, are feature-packed MTs that act like pocket-sized computers. They differ from traditional mobile phones that provide the function of making and receiving calls primarily. Internet access and mobile applications are essential elements that make MTs unique. Mobile applications are programs designed to work on a mobile device to provide various functions for specific purposes ( Wallace *et al.*, 2012), such as navigation, entertainment, or health care that supports daily life.

During the COVID-19 pandemic, engagement in online shopping, education, entertainment, and food delivery has become increasingly important ( Donthu & Gustafsson, 2020). MT provides the necessary channels to engage in activities during quarantine. Mobile applications contribute to managing the COVID-19 crisis by providing home monitoring, information sharing, contact tracing, and decision-making ( Kondylakis *et al.*, 2020). The COVID-19 pandemic has led to a phenomenon where many people have been forced to use MTs regardless of their preferences. Indeed, there are increasingly negative consequences of not using contemporary technologies in daily living. For instance, a person who does not use MT during COVID-19 will experience difficulties participating in social events, obtaining services, or interacting with others.

The nature of COVID-19 led to the phenomenon that middle-aged and older adults have a high risk of hospitalization rate and death rate after COVID-19 infection than younger people ( Centers for Disease Control and Prevention, 2023), which increases the essentiality of MT use to lower the risk of infection and health management among middle-aged and older adults. In addition, middle-aged and older adults tend to be less tech-savvy than younger. Younger adults aged 18 to 28 are likely to use a wider variety of technologies than adults aged 65 or older ( Olson *et al.*, 2011). Use of mobile health applications, for example, research indicated that adults aged <35 in the United States are more likely to use mobile health applications than adults aged ≥35 years old ( Bhuyan *et al.*, 2016). The phenomenon of younger adults having higher acceptance and technology usage than middle-aged and older groups has been evidenced in the previously developed Unified Theory of Acceptance and Use of Technology, which used “age” as a moderator for technology use and acceptance ( Venkatesh *et al.*, 2003). Hence, this study adopted the concept of potential age difference in MT use experience and focused on MT users aged 35 or older.

The threat of the SARS-CoV-2 virus undoubtedly has made MT a normal part of life. However, we have limited knowledge of how COVID-19 changed behavior, perception, and perspectives on using MT. Therefore, this cross-sectional study sought to document and compare MT use before and during COVID-19 among middle-aged and older adults in the United States. This study defines MTs as portable products that contain touchscreen functions, Internet access, and mobile applications. We hypothesized that the pandemic would lead to an increase in daily MT use, an increase in the perceived necessity of use, an increase in new MT functions adoptions, differences in the choice of MT functions used compared to before COVID-19, and trigger a shift of values regarding factors that affect MT use decisions and variation of perspectives toward MT.

## Methods

Inclusion criteria for this cross-sectional study were people who are: (1) aged 35 or older; (2) able to understand and communicate in English; (3) living in the United States; and (4) MT users. Participants were recruited via online survey portals, Amazon Mechanical Turk (MTurk), and Prolific (a free alternative could be the SurveyPlanet software). A snowball sampling strategy was used to increase the response rate of participants aged 65 or older by sharing online survey links and electronic flyers with potential participants using email and social media. The previously pilot-tested survey was distributed online on March 17, 2021, and the online survey response window closed on August 02, 2021.

### Instrument

At the pilot testing phase of survey development, responses from 17 female and 16 male respondents were included in the data analysis. None of the participants reported difficulty in accessing or finishing the survey. Ten participants gave positive feedback on the survey content. One participant had minor suggestions on the wording of the survey and added extra response options on questions regarding education level and reasons for adopting MT for the first time. The survey was refined based on the participants’ feedback.

The pilot-tested survey contained both closed-ended and open-ended questions. If the participant agreed to join the study and confirmed they met the inclusion criteria, a paragraph introducing MT was displayed to the participant. For questions about MT use before the pandemic, all participants were asked to answer the questions by recalling their experience before COVID-19.

There were four sections in the survey. Eleven questions collected data on participant characteristics and assessed the impact of COVID-19 on the individual’s life. Twelve questions collected information on participants’ experience with MT use before and during COVID-19; these included questions on the types of MT used, how long the participants had used MT, the main reason for using MTs, the frequency of using MTs each day, the top five most frequently used functions, and the top three factors that influence the decision to engage in MT use. Twenty-two statements (including 16 positive and six negative attitudes measured by a four-point Likert score) collected information about perspectives toward MT; these statements were adopted and modified from the extended Unified Theory of Acceptance and Use of Technology (UTAUT2), and studies aimed to examine MT acceptance and use ( Boontarig *et al.*, 2012; Shaw & Sergueeva, 2019; Venkatesh *et al.*, 2012). Finally, two open-ended questions encouraged respondents to comment on their responses in more depth. The list of the questions can be found in the Extended data ( Lin, 2023).

### Data cleaning

A total of 2,035 responses were recorded on the Qualtrics XM database (a free alternative could be the SoGoSurvey platform). Survey responses that met any of the following conditions were excluded from data analysis: Participants who did not meet the inclusion criteria (n=576), incomplete survey responses (participants who failed to read all the questions or skipped 50% of questions) (n=174), and unreliable survey responses (n=73). The strategies to identify unreliable survey responses included 1) participant spent less than three minutes on the survey; 2) duplicates or responses flagged as spam (when multiple identical responses were submitted from the same IP address within 12 hours) by the Qualtrics XM software; and 3) survey completed by a robot (the Qualtrics XM has adopted Google’s invisible reCaptcha technology for robot detection). After data cleaning, 1,212 usable survey responses were kept for analysis.

### Data analysis

Data analyses were completed using IBM SPSS version 28, which involved descriptive statistics demonstrating participant characteristics and Wilcoxon signed-rank test for the paired data that explored differences before and during COVID-19. Quotes collected from open-ended questions were used to support the findings and enrich the discussion and conclusion.

### Ethics approval

This study was approved by the University of Kentucky Medical Institutional Review Board (IRB) on Feb 25, 2021 (IRB number: 65430), and was performed in line with the principles of the Declaration of Helsinki.

### Participant consent

An IRB-approved electronic informed consent form was presented as the online survey cover letter and obtained from all participants included in the study. Each participant had to read the electronic consent and provide consent by ticking the box that represents agreement.

## Results

The median time to finish the survey is 13.3 minutes (interquartile range=8.6 minutes). As evidenced by the ZIP code, responses were received from a nationwide sample (20.5% from the West, 18.7% from the Midwest, 40.6% from the South, 17.7% from the Northeast region, and 2.4% did not provide a ZIP code). The average age of participants was 56 (ranging from 35 to 83). More than half (55.4%) were female. Most of the participants were white Caucasians (78.6%), were currently married (59.2%), had a college or higher education level (84.5%), and had annual incomes of USD 50,000 or higher (55.2%). More than three-quarters of the participants (77.9%) indicated good or excellent self-reported health status. With regard to residence, most were community-dwelling: 75.4% lived in a house, 21% in an apartment, and 1% in a retirement community. No participant lived in a skilled nursing facility.

Most of the participants (89.4%) owned two or more MT devices: 96.9% owned a smartphone, 74.8% owned an iPad or tablet, 32.3% owned a smartwatch, and 7.3% owned other MT devices, including a Kindle, laptop, and personal digital assistant. With regard to the length of use experience, 89.1% had used MT for more than three years, 5.4% for one to three years, 3.6% for more than six months but less than a year, and only 1.7% for six months or less. Responding to a question on the circumstances behind their initial decision to use MT, more than two-thirds (67.2%) indicated they needed it to function in today’s society, 39.5% adopted MT for its mobile applications, 38.2% because everyone around them was a user, and 26.8% due to job requirements. Finally, 9.8% stated other reasons that influenced their decision to adopt MT including “curiosity,” “safety,” “fun,” and “make extra money.”

During the pandemic, most participants (78.7%) reported having to stay in their residence more than before, 5.6% reported staying less, and 15.5% reported spending the same time. Two-thirds of the participants (66%) stated that they had less chance to go out due to the pandemic, 72.5% indicated having less opportunity to interact with people, and 33.3% spent more time staying with family due to the pandemic.

Regarding physical status, 25.4% reported having less chance to exercise during the pandemic. In contrast, 22.7% reported having more opportunities to exercise. Participants’ self-reported mental status indicated that 23.4% felt depressed, 34.9% felt anxious, and 38.9% felt stressed due to the pandemic. Finally, a few participants (7.9%) thought the pandemic did not affect their lives, and 18.6% felt their health was unaffected by the pandemic.

### Change in MT use behavior

MT daily use frequency during COVID-19 was significantly increased (z=-19.740, *p*<0.001) compared to before ( Table 1). Less than half of the participants (43.7%) spent more than 4 hours per day using MT before the pandemic, but this number increased to 68.2% during the pandemic.

**Table 1.** MT use frequency and perceived necessity to use.

Daily use frequency		
Frequency	Before the pandemic	During the pandemic
Less than an hour per day	N=145 (12%)	N=58 (4.8%)
About 1 to 3 hours per day	N=537 (44.3%)	N=325 (26.8%)
About 4 to 6 hours per day	N=296 (24.4%)	N=386 (31.8%)
About 7 to 9 hours per day	N=128 (10.6%)	N=257 (21.2%)
More than 9 hours per day	N=106 (8.7%)	N=184 (15.2%)
Missing values	N=0 (0%)	N=2 (0.2%)

Perceived necessity to use		
Necessity	Before the pandemic	During the pandemic
I do not feel the need to use MT in my daily life	N=85 (7%)	N=33 (2.7%)
I feel some need to use MT in my daily life	N=720 (59.4%)	N=283 (23.3%)
I feel strong need to use MT in my daily life	N=402 (33.2%)	N=891 (73.5%)
Missing values	N=5 (0.4%)	N=5 (0.4%)

MT: Mobile technology; N: Number of participants in the group; %: Percent of participants in the group.

All the participants provided information about their five most used MT functions before and during COVID-19 (ranked from most to least used). According to the ranks assigned by the participants to each function, the “text messaging” function was the most used before COVID-19, followed by “emails,” “voice calls/meetings,” “GPS/navigation,” “following breaking news,” and “playing games.” The most used MT functions during COVID-19 were “text messaging,” followed by “emails,” “voice calls/meetings,” “taxi/car service,” “online shopping,” and “video calls/meetings.” There was a significant rise (p<0.001) in the rank of harnessing the following MT functions during COVID-19 compared to before: video calls/meetings (percentage of participants who reported an increased rank: 29.3%, z=-12.160), online education (participants: 9.7%, z=-4.726), grocery/food delivery (participants: 22.0%, z=-11.083), online shopping (participants: 28.9%, z=-5.110), getting information about a health condition (participants: 11.6%, z=-5.652), and ordering taxi/car services (participants: 23.5%, z=-5.531).

In addition to use frequency and choice of functions, more than half of the participants (54.5%) reported experience using a new MT function during the pandemic. These included: online meetings, shopping (including delivery or pickup), health monitoring, receiving news updates, making reservations and checking-in, fitness programs, entertainment, and banking. In the open-ended section of the questionnaire, participants described their experience:

*“I now use curbside pickup and check-in using store apps, and I had never done that prior to the pandemic.”* (46-year-old female)

*“I have used a lot more the video calls function, either to communicate with my family and friends or for work. Online shopping became a necessity during the pandemic too.”* (36-year-old female)

*“I used mobile technology for online banking as it was not possible to go to a physical location for a long time.”* (65-year-old male)

### Change in MT use perception

There was a significant increase (z=-20.448, *p*<0.001) in the feeling of necessity to use MT during COVID-19 compared to before ( Table 1). Few participants (7.0%) did not feel the need to use MT in their daily life; this number decreased to 2.7% during the pandemic. One-third of the participants (33.2%) felt a strong need to use MT every day before COVID-19, and the number increased to 73.5% during COVID-19.

All the participants identified the top three factors (ranked from most to least important) that affected their decision to use MT before and during COVID-19. According to the ranks assigned by participants, the most crucial factor affecting the decision to use MT before COVID-19 was the “availability of functions that support daily life,” followed by the “necessity of using MT,” “ease of use,” and “pleasure of using MT.” On the other hand, the most crucial factor that affected the decision to use MT during COVID-19 was the “availability of functions that support daily life,” followed by the “necessity of using MT,” “social benefits of using MT,” and “ease of use.” There was a significant rise (p≤0.001) in considering the following factors essential in deciding to use MT during COVID-19 compared to before: physical benefits (percentage of participants who reported an increased rank: 14.9%, z=-4.440), social benefits (participants: 23.1%, z=-3.238), emotional benefits (participants: 18.3%, z=-6.343), the availability of functions that support daily life (participants: 29.3%, z=-4.265), and necessity (participants: 27.3%, z=-4.902).

### Change in perspectives toward MT

Participants’ agreement level to fifteen statements regarding positive perspectives toward MT significantly increased ( *p*<0.001) during COVID-19 compared to before ( Table 2). These statements reflected participants’ views about ease of use, the pleasure to use, and the benefits of using MT. Participants’ agreement level to one statement regarding positive view did not reach a significant difference ( *p*>0.05) compared between the time before and during COVID-19; the statement was “MTs are reasonably priced.”

**Table 2.** Change in perspectives toward MT.

Purpose	Perspectives	Decreased	Increased	Z score	P value
To measure the degree to which the participant believes that using MT will provide benefits in daily life.	MT is useful in improving my quality of life.	3.7%	26.5%	-13.315 ^b^	<0.001
	Using MT helps me to accomplish my job efficiently.	4.8%	23.6%	-10.836 ^b^	<0.001
	Using MT helps me accomplish daily tasks easily.	3.7%	23.6%	-12.282 ^b^	<0.001
To measure the degree of ease associated with the use of MT.	I find it easy to use MT for accessing services I need in daily living.	4.2%	18.8%	-9.934 ^b^	<0.001
	Learning to operate mobile applications is easy for me.	3.7%	11.3%	-6.246 ^b^	<0.001
To measure participant’s perceived pleasure derived from using MT.	Using the MT is enjoyable.	6.6%	14.2%	-5.178 ^b^	<0.001
	Using the MT is annoying.	10.7%	7.2%	-3.078 ^a^	0.002
To measure participant’s perceived emotional benefits (e), physical benefits (p), social benefits (sb), quality benefits (q), privacy risk (r), sacrifices (s) from using MT.	The quality of MT makes me want to use it. (q)	5.2%	14.2%	-5.504 ^b^	<0.001
	Using MT makes me feel relaxed. (e)	7.2%	16.5%	-5.607 ^b^	<0.001
	I have fear of using MT. (e)	9.9%	5.7%	-3.598 ^a^	<0.001
	MTs provide consistent quality in assisting daily tasks (shopping, traveling, etc.). (q)	4.6%	24.6%	-11.616 ^b^	<0.001
	I am worried that my privacy could be threatened due to the use of MT. (r)	5.4%	12.9%	-5.315 ^b^	<0.001
	My use of MT makes a good impression on other people. (sb)	4.5%	10.2%	-4.519 ^b^	<0.001
	Using MT would give its user social approval. (sb)	5.7%	12.3%	-5.029 ^b^	<0.001
	Using MT prevents me from developing other habits. (s)	8.1%	9.4%	-0.794 ^b^	0.427
	Using MT limits my human interaction in daily life. (s)	10.4%	19%	-4.821 ^b^	<0.001
	Using MT reduces the risk for me to get sick. (p)	4.8%	33.5%	-14.802 ^b^	<0.001
	Using MT helps me to maintain my physical health. (p)	5.9%	25.6%	-11.265 ^b^	<0.001
	MT plays a significant role in my daily life. (q)	4.2%	29.9%	-14.134 ^b^	<0.001
To measure participant’s perceived benefits received from the monetary cost.	MTs are reasonably priced.	7.4%	7%	-0.829 ^a^	0.407
	At the current price, MT provides good value.	7.6%	10.8%	-2.702 ^b^	0.007
	MTs are too expensive for me to use.	8.2%	7%	-1.630 ^a^	0.103

MT: Mobile technology.

Note: Analysis was conducted with the Wilcoxon signed ranks test; statements containing negative perspectives were marked underlined. The “decreased” and “increased” columns presented the percentage of participants that decreased or increased their agreement level with each statement during COVID-19 compared to before.

^a^ Based on positive ranks (agreement level during COVID-19 > before COVID-19).

^b^ Based on negative ranks (agreement level during COVID-19 < before COVID-19).

Participants’ agreement level with two statements regarding negative perspectives toward MT significantly decreased ( *p*<0.001) during COVID-19 compared to before. These statements reflected pleasure to use and emotional benefits, which were 1) “Using the MT is annoying;” and 2) “I have fear of using MT.” Two statements regarding negative views significantly increased ( *p*<0.001) during COVID-19 compared to before. These statements reflected perceived risk and perceived sacrifice of using MT, which were 1) “I am worried that my privacy could be threatened due to the use of MT;” and 2) “Using MT limits my human interaction in daily life.” Two statements regarding negative attitudes did not reach a significant difference ( *p*>0.05) between the time before and during COVID-19. These statements reflected the perceived sacrifice and perceived price value of MT, which were 1) “Using MT prevents me from developing other habits;” and 2) “MTs are too expensive for me to use.”

## Discussion

In this study, we have found a significant increase in daily use frequency and perceived necessity during COVID-19 compared to before. A significant increase was found in using several MT functions such as video calls/meetings, online education, grocery/food delivery, and ordering taxi/car services during COVID-19. More than half participants in this study had experience in adopting new MT functions during COVID-19. Participants increased their attention to the physical, social, and emotional benefits of using MT during COVID-19. Moreover, participants’ perspectives toward MT during COVID-19 positively increased.

People in at least 42 states were urged to stay home during the outbreak of COVID-19 ( Mervosh *et al.*, 2020). Evidence indicates that daily human mobility was reduced by 5% in the United States due to the stay-at-home policies ( Xiong *et al.*, 2020). In addition, previous studies found that the pandemic was associated with increased health anxiety and financial worry ( Tull *et al.*, 2020), led to significantly reduced physical activity ( Flanagan *et al.*, 2021; Knell *et al.*, 2020) or an increase of health-enhancing behavior due to having more time ( Knell *et al.*, 2020). Our study discovered changes in emotional status and time spent on physical activity during COVID-19, reinforcing previous studies’ findings.

Our study identified a significant increase in daily use frequency and perceived necessity to use during COVID-19. Similar results were found in a study in Jeju-si, South Korea (156 participants, age range: 15 to 80 years old), which reported increased MT use time due to COVID-19 ( Chae, 2020). Increased MT use frequency and perceived necessity suggested the potential influence of stay-at-home policies and spatial distancing strategies that were commonly applied during the pandemic.

Changes in commonly used functions of MT and the adoption of new functions during COVID-19 were discovered. Although text messaging, email, and voice calls were consistently the top three most used functions before and during COVID-19, people began to use more functions during COVID-19. These changes reflected different human needs for maintaining the quality of life during the pandemic. In-store shopping and meeting in person became very difficult during COVID-19, leading to the use and adoption of MT functions. Increased use of taxi/car services reflected people’s perception of the risk of using public transportation (e.g., bus) during the pandemic. As a result, demand for public transportation declined worldwide during the pandemic ( Tirachini & Cats, 2020). The change in behavior in using MT functions reflected adaptation to the situation in fulfilling daily tasks and transportation needs.

This study identified changes in prioritizing factors that influenced MT use decisions during COVID-19. The availability of functions that support daily life and the necessity of using MT were the top two factors before and during COVID-19, which reflected the common motivation to use MT. When MT becomes a requirement for living, people have no option but to adopt the technology to stay engaged. During COVID-19, people started to weigh the physical, social, and emotional benefits they could gain in deciding whether or not to use mobile devices. The finding suggested the importance of developing MT functions that create apparent benefits and introducing MT use benefits to users, which could be a solution for improving the quality of life in particular areas. For instance, a previous study demonstrated the benefits of telehealth coaching for patients who live in rural areas ( Young *et al.*, 2014). Based on our findings, healthcare delivery via a mobile device will be even more widely used in rural areas if the targeted users receive sufficient information regarding the benefits of remote health-promotion services.

Users’ perspectives toward MT became more positive in almost all dimensions during COVID-19 except the views of perceived risk and sacrifice. As for the price of mobile devices, people were likely to have similar perspectives toward it before and during COVID-19 since there was no remarkable adjustment in salary during the pandemic. The COVID-19 pandemic indeed created a once-in-a-lifetime platform to display the advantages of using MT; the effects were reflected in the changes in perspectives toward MT. However, we should not ignore that frequent use of MT might increase the privacy risk and prevent people from engaging in other in-person activities.

### Strengths and limitations

This study included the responses from participants of each region of the United States, which made our findings representative and reflected the MT use phenomenon in the United States. Our data cleaning process was strict and included multiple criteria to exclude unreliable data. Our study identified a shift of values regarding factors that affect MT use decisions, which offers the potential for developing strategies to increase MT adoption. The increase in MT adoption would provide a vast platform for delivering services to fulfill the variety of needs of MT users. Moreover, the findings of this study indicated a decrease in the variation of perspectives toward MT. We discovered substantial growth of acceptance toward MT during COVID-19; this is thus an excellent time to educate people about technology use and encourage teleservices development. Although the pandemic is less severe compared to the beginning of the outbreak and the COVID-19 restrictions are mostly lifted, the knowledge from this study in MT use during COVID-19 may serve to understand technology needs in this population better and to better plan for implementing new technologies.

Our study had several limitations—first, the lack of diversity in participants and recruitment methods. The data was collected during the severe period of the pandemic; thus, the recruitment method was extremely limited. Second, adding additional questions such as participants’ household arrangements, employment information, and the impact of COVID-19 on participants’ health would enrich the findings. Third, the survey study required participants to recall their experience with MT before COVID-19, which may lead to memory recall bias. Fourth, the survey question design could not consider personal factors (such as personality, intelligence, emotional state, and learning ability) that are more complex to measure. Fifth, this study might have response bias since the data was self-reported. Although the self-reported data had the advantage of revealing participants’ actual thoughts, participants’ concerns with social desirability might influence the data unconsciously.

Finally, it is essential to acknowledge that a shift in MT use behavior might already have occurred prior to the pandemic; COVID-19 might have simply provided a convenient excuse or trigger in accelerating behavior change. The data in this study do not allow us to explore MT use behavior changes before the pandemic, but such changes certainly occurred during the pandemic. More details about participant information and data on behavioral decisions during COVID-19 with respect to MT use would provide deeper insight.

### Future directions

This study could help design future studies to examine the relationships between health, technology implementations, acceptance, and adoption among middle-aged and older adults in the United States during the continuous waves of pandemic and post-pandemic. Comprehensive knowledge regarding the associations between MT use behavior, physical health, and mental health warrant longitudinal studies in the future. MT use could become even more critical in maintaining the quality of life; thus, future studies could consider recruiting diverse participant groups, including non-technology users, to explore MT use and adoption. Providers working on service delivery via mobile devices should explore strategies for increasing learning motivation. Developing strategies to give people a comfortable experience while embracing technology and making people in diverse situations learn fast will be more critical than ever. A 56-year-old female participant in the study explained, “ *I worry that as I get older, the world will depend more on mobile technology, and my ability to use it will decline.*” A 63-year-old female who described her experience of using MT noted, “ *The technologies change so quickly, that I gave up on trying to keep up with all of them.*” Technology use is supposed to bring convenience and efficiency to human beings; it is essential to reduce the burden of people trying to use it. Following the rapid steps of technology evolution, the question of how to provide a friendly interface or strategy for human-machine interaction demands immediate attention.

## Conclusion

The environment under the influence of COVID-19 has increased MT use behavior, acceptance, and mobile application adoption in the United States. The knowledge gained from this study will have implications to help remove barriers to using and accepting MT and provide directions for MT development and implementation in middle-aged and older populations.

## Data Availability

Anonymized data from this study are available on request from the corresponding author [YL] from the date of publication. The anonymized data are for academic use only; the request should contain a full research plan. The data are not publicly available as they contain information that could compromise research participant consent. Figshare: Questionnaire for Mobile Technology Use during COVID-19, https://doi.org/10.6084/m9.figshare.23613828.v1 ( Lin, 2023). This project contains the following extended data:
- Survey questions for investigating mobile technology use behavior during COVID-19 Data are available under the terms of the Creative Commons Attribution 4.0 International license (CC-BY 4.0).
