# Supplementary material for: Impact of COVID-19 on Mobile Technology use in adults in the United States
Source: F1000Res. 2023 Oct 18;12:1376. [Version 1] doi: 10.12688/f1000research.136724.1 (PMC12397735; doi:10.12688/f1000research.136724.1)
Supplement: Peer review history

# Reviewer report: Reviewer response for version 1

**DOI:** 10.5256/f1000research.149868.r400688

**Published:** 2025-08-29

**Authors:** ANUSUYAH SUBBARAO

**Affiliations:** 1 Multimedia University, Cyberjaya, Malaysia

1. Is the work clearly and accurately presented and does it cite the current literature?

 The article is well-structured and clearly written. The introduction effectively contextualizes the research within existing literature on COVID-19's impact on digital technology adoption. The authors appropriately cite current literature, including relevant studies on pandemic-related technology use, the Unified Theory of Acceptance and Use of Technology (UTAUT2), and mobile health applications. The writing is accessible while maintaining scientific rigor, and the progression from introduction through methods to results and discussion is logical and coherent.

 2. Is the study design appropriate and is the work technically sound?

 The cross-sectional design is appropriate for documenting changes in MT use during a specific time period. The study addresses an important and timely research question about technology adoption during a global crisis. The focus on adults aged 35 and older is well-justified given that this population typically shows lower technology adoption rates and faced higher COVID-19 health risks. The inclusion criteria are clearly defined and appropriate for the research objectives.

 3. Are sufficient details of methods and analysis provided to allow replication by others?

 The methodology section provides comprehensive details enabling replication:

- Clear inclusion criteria and recruitment strategies
- Detailed description of the pilot testing process with 33 participants
- Thorough explanation of the four-section survey structure
- Specific data cleaning procedures with exclusion criteria
- Statistical analysis methods (descriptive statistics and Wilcoxon signed-rank tests)
- Sample size and demographic characteristics
- Ethical approval information

The authors also mention that the full questionnaire is available in extended data, which enhances reproducibility.

 4. Is the statistical analysis and its interpretation appropriate?

 The statistical approach is sound and appropriate for the research design:

- Wilcoxon signed-rank tests are correctly used for paired, non-parametric data comparing before and during COVID-19 responses
- Descriptive statistics appropriately characterize the sample
- The presentation of results includes both statistical significance (p-values, z-scores) and practical significance (percentages of participants showing changes)
- The authors appropriately report effect sizes through percentages of participants experiencing increases or decreases
- The interpretation of statistical results is conservative and accurate

5. Are all the source data underlying the results available to ensure full reproducibility?

 Partly

 While the authors state that "anonymized data from this study are available on request from the corresponding author," this creates a potential barrier to full reproducibility. The data are restricted to "academic use only" and require "a full research plan," which may limit accessibility for verification purposes. However, the authors provide reasonable justification for this approach, citing participant consent considerations. The extended data (questionnaire) is publicly available, which partially addresses reproducibility concerns.

 6. Are the conclusions drawn adequately supported by the results?

 The conclusions are well-supported by the presented data:

- Statistical evidence clearly supports claims about increased MT use frequency and perceived necessity
- The identification of specific function usage changes (video calls, online education, delivery services) is backed by significant statistical findings
- The discussion appropriately contextualizes findings within the broader literature
- Limitations are honestly acknowledged and discussed
- The implications for MT development and implementation are reasonable extrapolations from the findings

Minor Suggestions:

1. Consider discussing potential strategies to validate recalled pre-pandemic behavior in future studies
2. The discussion could benefit from more specific recommendations for technology developers and policymakers
3. Consider expanding the limitations section to address potential selection bias from online recruitment methods

Overall Assessment:

 This is a well-executed study that makes a valuable contribution to understanding technology adoption during crisis periods. The methodology is sound, the analysis is appropriate, and the findings are clearly presented and well-supported. While there are inherent limitations in the retrospective design, the authors acknowledge these honestly and the study provides important insights for both researchers and practitioners interested in technology adoption among middle-aged and older adults.

Is the work clearly and accurately presented and does it cite the current literature?

Yes

If applicable, is the statistical analysis and its interpretation appropriate?

Yes

Are all the source data underlying the results available to ensure full reproducibility?

Partly

Is the study design appropriate and is the work technically sound?

Yes

Are the conclusions drawn adequately supported by the results?

Yes

Are sufficient details of methods and analysis provided to allow replication by others?

Yes

Reviewer Expertise:

Digital Transformation, Software Engineering,

I confirm that I have read this submission and believe that I have an appropriate level of expertise to confirm that it is of an acceptable scientific standard.

# Reviewer report: Reviewer response for version 1

**DOI:** 10.5256/f1000research.149868.r219469

**Published:** 2023-12-21

**Authors:** Sri Rahayu Natasia

**Affiliations:** 1 Lecturer of Information Systems Department, Institut Teknologi Kalimantan, Balikpapan, East Kalimantan, Indonesia

I think this paper is superb, I enjoyed reading it. The overall structure is coherent and well-explained. The authors explain the introduction part clearly and refer to the latest publications as they talk about the phenomena of MT use during COVID-19. The method section, instrument, and data cleaning are excellent for producing high-quality and reliable data for further analysis. This paper discovers changes in MT use behavior, MT use perception, and perspective toward MT on users before and during the pandemic COVID-19 in the USA. Strengths and limitations, also future directions of this research are insightful to the reader and potential researcher on a similar topic.

Is the work clearly and accurately presented and does it cite the current literature?

Yes

If applicable, is the statistical analysis and its interpretation appropriate?

I cannot comment. A qualified statistician is required.

Are all the source data underlying the results available to ensure full reproducibility?

Yes

Is the study design appropriate and is the work technically sound?

Yes

Are the conclusions drawn adequately supported by the results?

Yes

Are sufficient details of methods and analysis provided to allow replication by others?

Yes

Reviewer Expertise:

Information System Evaluation, Technology Acceptance, UI/UX Research

I confirm that I have read this submission and believe that I have an appropriate level of expertise to confirm that it is of an acceptable scientific standard.
